# DPEP1 promotes drug resistance in colon cancer cells by forming a positive feedback loop with ASCL2

**DOI:** 10.1002/cam4.4926

**Published:** 2022-06-06

**Authors:** Cheng Zeng, Guoping Qi, Ying Shen, Wenjing Li, Qi Zhu, Chunxia Yang, Jianzhong Deng, Wenbin Lu, Qian Liu, Jianhua Jin

**Affiliations:** ^1^ Department of Oncology Wujin Hospital Affiliated with Jiangsu University Changzhou Jiangsu Province China; ^2^ Department of Oncology Wujin Clinical College of Xuzhou Medical University Changzhou Jiangsu Province China

**Keywords:** ASCL2, bioinformatics, colon cancer, DPEP1, drug resistance

## Abstract

**Background:**

Drug resistance is an important factor affecting the efficacy of chemotherapy in patients with colon cancer. However, clinical markers for diagnosing drug resistance of tumor cells are not only a few in number, but also low in specificity, and the mechanism of action of tumor cell drug resistance remains unclear.

**Methods:**

Dipeptidase 1 (DPEP1) expression was analyzed using the cancer genome atlas (TCGA) and genotype‐Tissue Expression pan‐cancer data. Survival analysis was performed using the survival package in R software to assess the prognostic value of DPEP1 expression in colon cancer. Correlation and Venn analyses were adopted to identify key genes. Immunohistochemistry, western blot, qRT–PCR, Co‐immunoprecipitation, and dual‐luciferase reporter experiments were carried out to explore the underlying associations between DPEP1 and Achaete scute‐like 2 (ASCL2). MTT assays were used to evaluate the role of DPEP1 and ASCL2 in colon cancer drug resistance.

**Results:**

DPEP1 was highly expressed in colon cancer tissues. DPEP1 expression correlated negatively with disease‐specific survival but not with overall survival. Bioinformatics analysis and experiments showed that the expressions of DPEP1 and ASCL2 in colon cancer tissues were markedly positively correlated. Mechanistic research indicated that DPEP1 enhanced the stability of protein ASCL2 by inhibiting its ubiquitination‐mediated degradation. In turn, ASCL2 functioned as a transcription factor to activate the transcriptional activity of the DPEP1 gene and boost its expression. Furthermore, DPEP1 also could enhance the expression of colon cancer stem cell markers (LGR5, CD133, and CD44), which strengthened the tolerance of colon cancer cells to chemotherapy drugs.

**Conclusions:**

Our findings reveal that the DPEP1 enhances the stemness of tumor cells by forming a positive feedback loop with ASCL2 to improve resistance to chemotherapy drugs.

## Graphical abstract

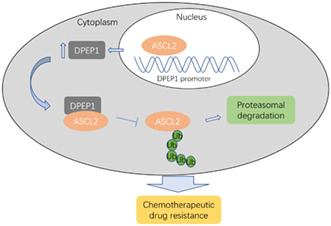

## 1 INTRODUCTION

Colorectal cancer is a common malignant tumor in the digestive system. Its global incidence rate ranks third, and its mortality rate ranks second in cancer‐related deaths. Drug resistance is an important factor affecting the efficacy of chemotherapy in patients with colon cancer.^1^ However, clinical markers for diagnosing drug resistance of tumor cells are not only a few in number, but also low in specificity. Moreover, the mechanism of action of tumor cell drug resistance remains unclear. With the rapid development of high‐throughput sequencing and gene chip technology, bioinformatics methods have become increasingly crucial for analyzing massive genetic data. An increasing number of driver genes have been discovered.^2^ However, drug resistance gene screening and related mechanism research need further improvement.

Dipeptidase 1 (DPEP1) is a zinc‐dependent metalloproteinase that participates in antibiotic processing, dipeptide hydrolysis, and glutathione and leukotriene metabolism.^3^, ^4^ Recently, additional attention has been given to the effect of DPEP1 on cancers. Studies have shown that DPEP1 can promote hepatoblastoma progression and leukemia cell proliferation.^5^, ^6^ In colorectal cancer, DPEP1 expression is upregulated, the DPEP1 high‐expression group shows a poor prognosis,^7^ and DPEP1 is considered a specific tumor marker for colon cancer.^8^ Moreover, DPEP1 promotes colon cancer cell proliferation, metastasis, and invasion.^9^, ^10^, ^11^ Recently, single‐cell RNA sequencing indicated that DPEP1 plays a key role in the evolution from ulcerative colitis to ulcerative colitis‐associated colon cancer.^12^ However, the underlying mechanism of DPEP1 in colon cancer drug resistance remains elusive.

Achaete scute‐like 2 (ASCL2), a downstream target of the Wnt/β‐catenin signaling pathway, is a helix–loop–helix transcription factor that is overexpressed in colorectal cancer and is considered an oncogene that promotes proliferation and liver metastasis.^13^, ^14^, ^15^ Moreover, ASCL2 could inhibit apoptosis and increase resistance of cancer cells to chemotherapy drugs.^16^, ^17^, ^18^ It was also reported that ASCL2 promotes epithelial‐mesenchymal transition induced by HIF‐1α overexpression.^19^ Importantly, ASCL2 plays an indispensable role in LGR5^+^ intestinal stem cells and colorectal cancer progenitor cells.^20^, ^21^, ^22^, ^23^

In this study, colon cancer data in the the cancer genome atlas (TCGA) and GEO databases were analyzed through bioinformatics analysis, and DPEP1 was found to be highly expressed in colon cancer. And DPEP1 expression correlated with poor disease‐specific survival (DSS) in colon cancer patients. Interestingly, we found that ASCL2 expression was significantly positively correlated with that of DPEP1 in colon cancer tissues. In‐depth study, we found that DPEP1 enhanced the protein stability of ASCL2 by inhibiting its ubiquitin‐mediated degradation. In turn, ASCL2 functioned as a transcription factor to activate the transcriptional activity of the DPEP1 gene and enhance its expression. Moreover, DPEP1 also increased markedly other stem cells markers, such as LGR5, CD133, and CD44. Functionally, DPEP1 expression increased the resistance of colon cancer cells to the chemotherapy drugs oxaliplatin and irinotecan in the ASCL2‐dependent manner. In this way, DPEP1 and ASCL2 formed a positive feedback loop regulation mode, which increased the tolerance of tumor cells to chemotherapeutic drugs. Thus, this study will provide a potentially important colon tumor drug resistance marker for clinical practice, and this positive feedback loop regulation will also provide a new perspective for tumor research.

## 2 MATERIALS AND METHODS

### 2.1 Data collection

We downloaded the pan‐cancer transcriptome data in the TCGA database and the normal tissue transcriptome data in the Genotype‐Tissue Expression (GTEx) database from the University of California, Santa Cruz (UCSC) website (https://xenabrowser.net/datapages/). The data format is fragments per kilobase million. The expression level of DPEP1 in pan‐cancer was analyzed after merging the expression profiling data of the above two databases. GSE74602, based on the GPL6104 platform, was downloaded from the GEO database, including 30 paired normal and tumor colon tissues.

### 2.2 Survival analysis

Survival analysis was performed using the survival package in R software (version 4.0.2) to assess the prognostic value of DPEP1 expression in colon cancer.^24^ Samples were divided into a high‐expression group and a low‐expression group with the best cutoff value for DPEP1 mRNA expression in colon cancer, and the overall survival (OS) and DSS of the different expression groups were compared. Survival curves were plotted using the Kaplan–Meier method. Cox regression was used to evaluate statistical significance. *p* < 0.05 was considered significant.

### 2.3 Correlation, DEGs, and Venn analysis

Pearson's correlation analysis of DPEP1 mRNA and other mRNAs in colon cancer was performed using TCGA COAD data. Differentially expressed genes (DEGs) were obtained using the limma package in R software.^25^ The false‐positive results could be corrected by the adjusted *p* value (adj. *p* value) of the false discovery rate method.^26^ The cutoff criteria for DEGs were as follows: adjusted *p* < 0.05 and | log_2_‐fold change (FC)| > 1.5. The top 100 genes that were most positively associated with DPEP1 obtained from correlation analysis, upregulated genes in TCGA‐COAD, and upregulated genes in GSE74602 were selected for Venn analysis.

### 2.4 Cell lines and tissue samples

Colon cancer cell lines, including HCT116, SW480, SW620, and RKO cells, were purchased from the Cell Bank at the Shanghai Institute of Cells, Chinese Academy of Sciences. Cells were cultured in DMEM, or 1640 medium (Gibco) supplemented with 10% fetal bovine serum (Sangon Biotech) and 1% penicillin/streptomycin (Beyotime) at 37°C with 5% CO_2_. Forty pairs of colon cancer and adjacent normal tissues were obtained from Wujin Hospital affiliated with Jiangsu University. Informed consent was obtained from patients. This study was approved by the Ethics Committee of Wujin Hospital affiliated with Jiangsu University.

### 2.5 Plasmids, siRNAs, and cell transfection

Plasmids, including pCMV, pCMV‐DPEP1, pcDNA3, and pcDNA3‐ASCL2, were constructed using the MiaoLing Plasmid Sharing Platform (MiaolingBio). All siRNAs and related primer sequences are listed in Table S1. According to the manufacturer's protocol, cell transfection using TurboFect Transfection (Thermo Fisher Scientific) was conducted as described previously.^27^ Briefly, cells were seeded in six‐well plates (Corning), grown to a cell density of 70%, and then transfected with pCMV (0.8 μg), pCMV‐DPEP1 (0.8 μg), pcDNA3 (0.8 μg), or pcDNA3‐ASCL2 (0.8 μg) and cultured at 37°C with 5% CO_2_ for 48 h. Western blotting and qRT–PCR were used to test transfection efficiency following cell collection.

### 2.6 RNA extraction and qRT–PCR

Total RNA was extracted from cells using TRIzol reagent (TaKaRa) according to the manufacturer's protocol. A total of 1000 μg of RNA was used for first‐strand cDNA synthesis using a TaqMan cDNA synthesis kit (TaKaRa). qRT–PCR was conducted using a SYBR Green PCR Kit (TaKaRa) on a 7500 Real‐time PCR System (TaKaRa). The detailed procedures were carried out as previously described.^9^ The primer sequences are listed in Table S1. The 2^−ΔΔCt^ method was used to calculate the relative levels.

### 2.7 Immunohistochemical staining

Immunohistochemical staining assays were performed according to a published paper.^28^ Normal colon tissues and colon cancer tissues were blocked in 5% bovine serum albumin for 30 min at room temperature and then incubated with DPEP1 (dilution: 1:100, Abcam) or ASCL2 (dilution: 1:100, Abcam) primary antibodies at 4°C overnight, followed by incubation with a secondary antibody conjugated with horseradish peroxidase (dilution: 1:100; Sangon Biotech) at room temperature for 60 min. Immunostaining was conducted using a diaminobenzidine substrate kit (Sangon Biotech) and counterstained with hematoxylin. Images were obtained using an inverted microscope (magnification, 200×).

### 2.8 Western blotting

The cells were washed with PBS, and the proteins were extracted using RIPA lysis buffer (Sangon Biotech). Proteins were segregated using 8% SDS–PAGE and transferred to PVDF membranes (Millipore). The PVDF membranes were then incubated with DPEP1 (dilution: 1:1000, Abcam), ASCL2 (dilution: 1:1000, Abcam), β‐actin antibody (dilution: 1:4000, Sangon Biotech) or ubiquitin (dilution: 1:1000, Boston Biochem) after incubation with goat anti‐rabbit or goat anti‐mouse IgG HRP antibody (dilution: 1:5000, Sangon Biotech). The bands were measured using an ECL detection kit (Labgic). The gray analysis for the protein bands was performed using ImageJ software.

### 2.9 Co‐immunoprecipitation assays

HCT116 and SW620 cells were collected and lysed in immunoprecipitation cell lysis buffer (Sangon Biotech). The lysates were incubated with DPEP1 (Abcam) or ASCL2 (Abcam) primary antibodies at 4°C for 4 h and then incubated with protein A/G‐agarose beads (Sangon Biotech) at 4°C overnight. After three washes, immunoprecipitated samples were subjected to SDS–PAGE followed by western blotting with the indicated antibodies. The detailed procedures were described previously.^28^

### 2.10 ASCL2 stability experiments

HCT116 cells were transiently transfected with pCMV (0.8 μg) or pCMV‐DPEP1 (0.8 μg) in six‐well plates. SW620 cells were transiently transfected with siRNA (100 nM) or DPEP1 siRNA (100 nM). After 48 h, the samples were treated with cycloheximide (CHX, Sigma) at a final concentration of 15 mg/ml for 0, 0.5, and 1.0 h. ASCL2 protein levels were tested by western blotting, and then the ASCL2 density normalized to β‐actin was plotted using ImageJ and GraphPad Prism software. The detailed procedures followed as previously described methods.^9^

### 2.11 Protein degradation assays

SW620 cells were transiently transfected with siRNA (100 nM) or DPEP1 siRNA (100 nM) for 48 h and then treated with 10 mM proteasome inhibitor MG132 for 6 h. Subsequently, the cells were lysed on ice for 30 min and centrifuged at 12,000 *g* for 20 min. Lysates were incubated with ASCL2 primary antibody for 4 h at 4°C, and then ASCL2 protein was immunoprecipitated with protein A/G‐agarose beads from cell lysates at 4°C overnight. The ubiquitylation level of ASCL2 protein was detected by western blotting with an anti‐ubiquitin antibody. The detailed procedures followed as previously described methods.^9^

### 2.12 Dual‐luciferase reporter experiments

To test the transcriptional activity of the DPEP1 promoter, we constructed the DPEP1 promoter (−1500 bp/+200 bp region) into pGL3‐Basic plasmids and then tested the activity of the pGL3‐DPEP1 promoter by dual‐luciferase reporter experiments. Dual‐luciferase reporter experiments were performed as previously described.^27^

### 2.13 Chemotherapy drug killing experiment

pCMV, pCMV‐DPEP1, and pCMV‐DPEP1 + siASCL2 were transiently transfected into HCT116 or RKO cells. After 48 h, cells from each group were seeded in 96‐well plates at a density of 5000 cells per well and incubated at 37°C overnight. The culture medium was replaced with a fresh culture medium with different concentrations of oxaliplatin (0, 40, and 80 μM) or irinotecan (0, 5, and 10 μM) with five replicates each. After a 48 h incubation, 20 μl of MTT (5 mg/ml) was added to each well and incubated at 37°C for 4 h. Subsequently, the culture medium was removed, and 150 μl of DMSO was added to each well. After shaking for 10 min, the 96‐well plate was read on an enzyme‐labeled device at 490 nm to calculate the cell viability rate.

### 2.14 Statistical analysis

All results are shown as the mean ± standard deviation. GraphPad Prism 8 software was used to conduct statistical analyses. Student's *t*‐test or one‐way analysis of variance was used to determine significant differences. *p* < 0.05 (*), *p* < 0.01 (**), and *p* < 0.001 (***) were considered significant. All experiments were repeated at least three times independently.

## 3 RESULTS

### 3.1 DPEP1 is highly expressed in COAD

**FIGURE 1:**
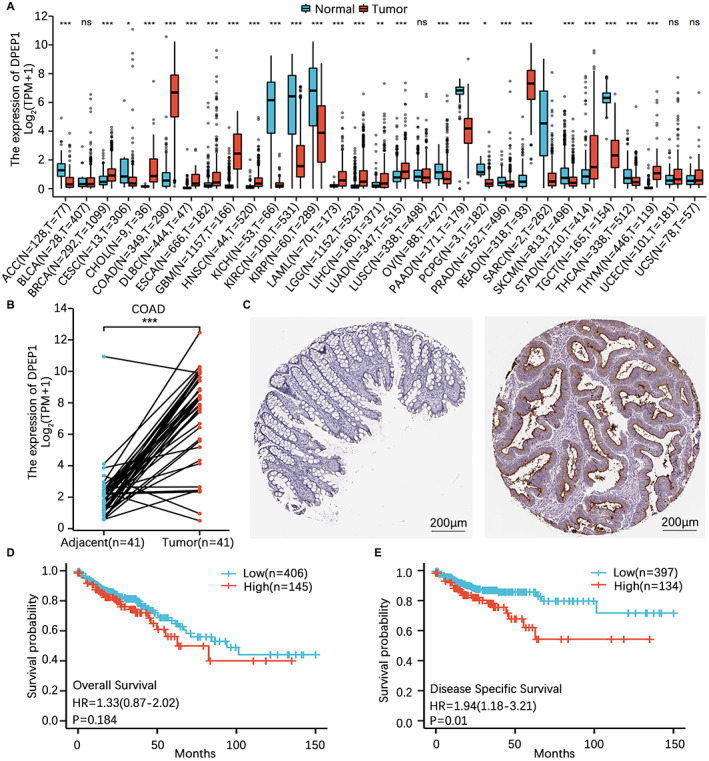
DPEP1 is highly expressed in colon cancer. (A) DPEP1 expression in tumor and normal tissues in TCGA and GTEx pan‐cancer data. (B) DPEP1 expression in paired normal and tumor tissues in COAD from TCGA. (C) Immunohistochemical image showing DPEP1 expression in normal colon tissues and colon cancer tissues from the Human Protein Atlas. The correlation between DPEP1 expression and overall survival (D) and disease‐specific survival (E) in colon cancer was analyzed using the TCGA database with the best cutoff value for DPEP1 mRNA expression. *p < 0.05; **p < 0.01; ***p < 0.001; ns indicates no statistical significance. The data are shown as the mean ± SD. DPEP1, dipeptidase 1; GTEx, genotype‐tissue expression; TCGA, the cancer genome atlas.

To better understand the possible role of DPEP1 in colon cancer tumors, we first explored the expression levels of DPEP1 in pan‐cancer tissues, DPEP1 expression data from TCGA and GTEx databases were analyzed. As shown in Figure 1A, DPEP1 was highly expressed in BRCA, CHOL, COAD, DLBC, ESCA, GBM, HNSC, LAML, LGG, LIHC, LUAD, READ, STAD, and THYM carcinoma tissues (Table S2). In 41 pairs of colon cancer tissues and adjacent normal tissues, DPEP1 was markedly highly expressed in cancer tissues (Figure 1B). Immunohistochemistry images downloaded from The Human Protein Atlas database further confirmed that DPEP1 was highly expressed in colon cancer tissues (Figure 1C). Bioinformatics analysis indicated that DPEP1 expression was correlated negatively with DSS (Figure 1E) but did not correlate with OS (Figure 1D). Moreover, survival analysis for DPEP1 in pan‐cancer was performed with the best cutoff value for DPEP1 mRNA expression. The results showed that DPEP1 expression levels in ACC, BLCA, LGG, SARC, STAD, and UCS were associated with unfavorable OS, while HNSC, KIRC, KIRP, SKCM, and THYM patients with high expression of DPEP1 had better OS (Figure S1). Collectively, colon cancer tissues expressed high levels of DPEP1, which was negatively correlated with DSS in colon cancer patients.

### 3.2 Screening of DPEP1 gene expression‐related factors in colon cancer

**FIGURE 2:**
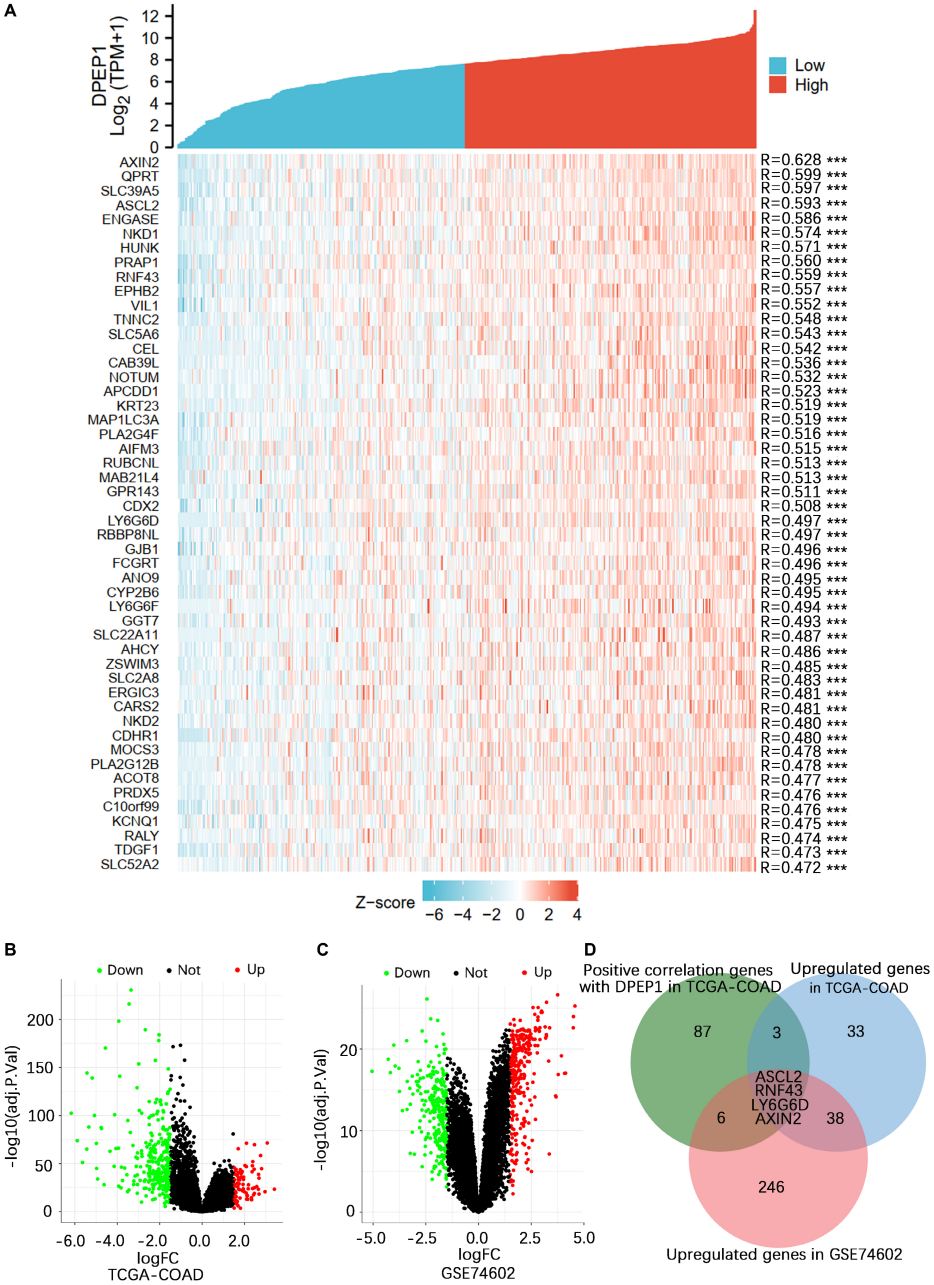
Screening of DPEP1 gene expression‐related factors in colon cancer. (A) The top 50 genes most positively associated with DPEP1 were shown in a heatmap. The data were normalized using the *Z* score standardization method. ***p < 0.001. Volcano plots of the distribution of DEGs in TCGA‐COAD (B) and the GSE74602 dataset (C). (D) Venn analysis of the upregulated genes in TCGA‐COAD, the upregulated genes in GSE74602, and the top 100 most positively correlating genes with DPEP1 in TCGA‐COAD. DEG, differentially expressed genes; DPEP1, dipeptidase 1; TCGA, the cancer genome atlas.

To screen factors associated with DPEP1 gene expression in colon cancer tissues, transcriptome data downloaded from the TCGA database (Table S3) were subjected to Pearson correlation analysis, 50 most positively associated genes with DPEP1 are shown in a heatmap (Figure 2A). The gene expression data from the TCGA‐COAD database and GSE74602 dataset were subjected respectively to differential expression gene analysis with an adjusted *p* value <0.05 and | log_2_ FC| > 1.5 (Figure 2B,C; Tables S4 and S5). The top 100 genes that were most positively associated with DPEP1 obtained from correlation analysis, upregulated genes in TCGA‐COAD, and upregulated genes in GSE74602 were selected for Venn analysis, four core genes (ASCL2, RNF43, LY6G6D, and AXIN2) were obtained (Figure 2D; Table S6). Combining the two factors of high correlation and significantly high expression in colon cancer, ASCL2 was selected for the correlation study with DPEP1.

### 3.3 The stability of ASCL2 protein was maintained by DPEP1 in colon cells

**FIGURE 3:**
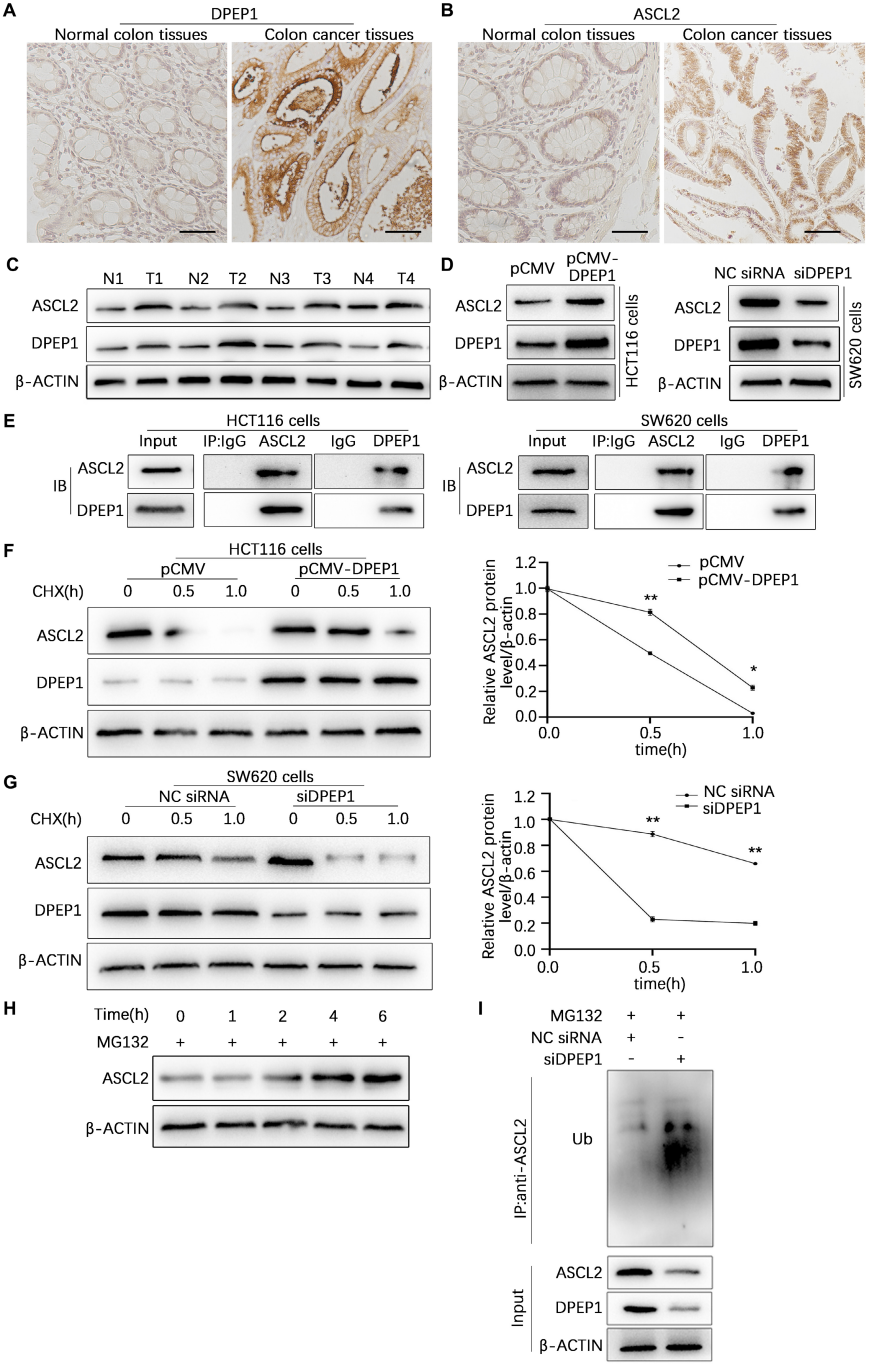
ASCL2 protein stability was maintained by DPEP1 in colon cells. Immunohistochemical analysis of DPEP1 (A) and ASCL2 (B) in normal colon tissues and colon cancer tissues. (C) Western blot analysis of DPEP1 and ASCL2 in normal colon and colon cancer tissues. (D) Western blot analysis of DPEP1 and ASCL2 in HCT116 cells and SW620 cells. (E) Reciprocal CoIP analysis of DPEP1 and ASCL2 proteins in HCT116 and SW620 cells. (F) ASCL2 protein levels in HCT116 cells transiently transfected with pCMV (0.8 μg) or pCMV‐DPEP1 (0.8 μg) plasmids treated with CHX (15 mg/ml) for 0, 0.5, and 1.0 h were evaluated by western blot. The ASCL2 intensity normalized to β‐actin was plotted. (G) ASCL2 protein levels in SW620 cells transiently transfected with NC siRNA or DPEP1 siRNA (100 nM) treated with CHX (15 mg/ml) for 0, 0.5, and 1.0 h were evaluated by western blot. The ASCL2 intensity normalized to β‐actin was plotted. (H) ASCL2 protein levels in HCT116 cells treated with the proteasome inhibitor MG132 (10 mM) for 0, 1, 2, 4, or 6 h were measured by western blot. (I) SW620 cells were transfected with NC siRNA or DPEP1 siRNA (100 nM) for 48 h and then treated with 10 mM proteasome inhibitor MG132 for 6 h. ASCL2 protein was immunoprecipitated with protein A/G‐agarose beads from cell lysates, and then the ubiquitylation level of ASCL2 protein was detected by western blotting with an anti‐ubiquitin antibody. ASCL2, Achaete scute‐like 2; CHX, cycloheximide; CoIP, co‐immunoprecipitation; DPEP1, dipeptidase 1; NC, negative control. * represents *p* < 0.05; ** represents *p* < 0.01.

Bioinformatics analysis showed that ASCL2 and DPEP1 were both markedly expressed in colon cancer tissues and had a significantly positive correlation. To verify the accuracy of the bioinformatics analysis results, immunohistochemistry, and western blotting assays were conducted to validate DPEP1 and ASCL2 protein levels in normal colon tissues and colon cancer tissues, respectively. The results demonstrated that both DPEP1 and ASCL2 were highly expressed in colon cancer tissues compared to normal tissues (Figure 3A–C). There was a significantly positive correlation between the expression of DPEP1 and ASCL2 in colon cancer, according to which we speculated that there was a mutual regulatory relationship between them. We then transfected HCT116 cells with pCMV or pCMV‐DPEP1, respectively, and the results indicated that overexpression of DPEP1 resulted in a significant increase in the expression of ASCL2 colon cancer cells (Figure 3D). While SW620 cells transfected with negative control (NC) siRNA or siDPEP1 were measured by western blotting, which indicated that knockdown of DPEP1 also markedly reduced the expression of ASCL2 (Figure 3D). Moreover, Co‐immunoprecipitation experiments showed that DPEP1 and ASCL2 proteins could bind to each other in HCT116 and SW620 cells (Figure 3E). Because our previous study found that DPEP1 did not significantly regulate ASCL2 at the transcriptional level, we hypothesized that DPEP1 held the stability of ASCL2 at the protein level. HCT116 cells and SW620 cells were transfected respectively with pCMV‐DPEP1 or siDPEP1 for 48 h, the cells were then treated with CHX (a protein synthesis inhibitor) for different time periods, and the degradation rate constant of ASCL2 protein was analyzed by western blotting. Compared with the control groups, DPEP1 overexpression in HCT116 cells lengthened the half‐life of the ASCL2 protein, whereas DPEP1 interference in SW620 cells accelerated ASCL2 degradation (Figure 3F,G). MG132 (a proteasome inhibitor) was used to inhibit the ubiquitin‐proteasomal degradation pathway in cells. HCT116 cells treated with MG132 for 0, 1, 2, 4, or 6 h were then measured to evaluate ASCL2 protein levels by western blotting, which showed that the protein levels of ASCL2 in cells increased with MG132 treatment (Figure 3H), suggesting that ASCL2 protein was degraded by the ubiquitination pathway of the proteasome. To investigate whether ASCL2 ubiquitination was regulated by DPEP1, immunoprecipitation was used to pull down ASCL2 proteins. Subsequently, the ubiquitination levels of ASCL2 proteins were evaluated by western blotting using an anti‐ubiquitin antibody. The results indicated that DPEP1 knockdown in SW620 cells increased the levels of polyubiquitinated ASCL2 (Figure 3I). In summary, DPEP1 binds to ASCL2 in colon cancer cells and enhances protein ASCL2 stability by inhibiting its ubiquitin‐proteasomal degradation.

### 3.4 ASCL2 increased DPEP1 expression by activating its transcriptional activity

**FIGURE 4:**
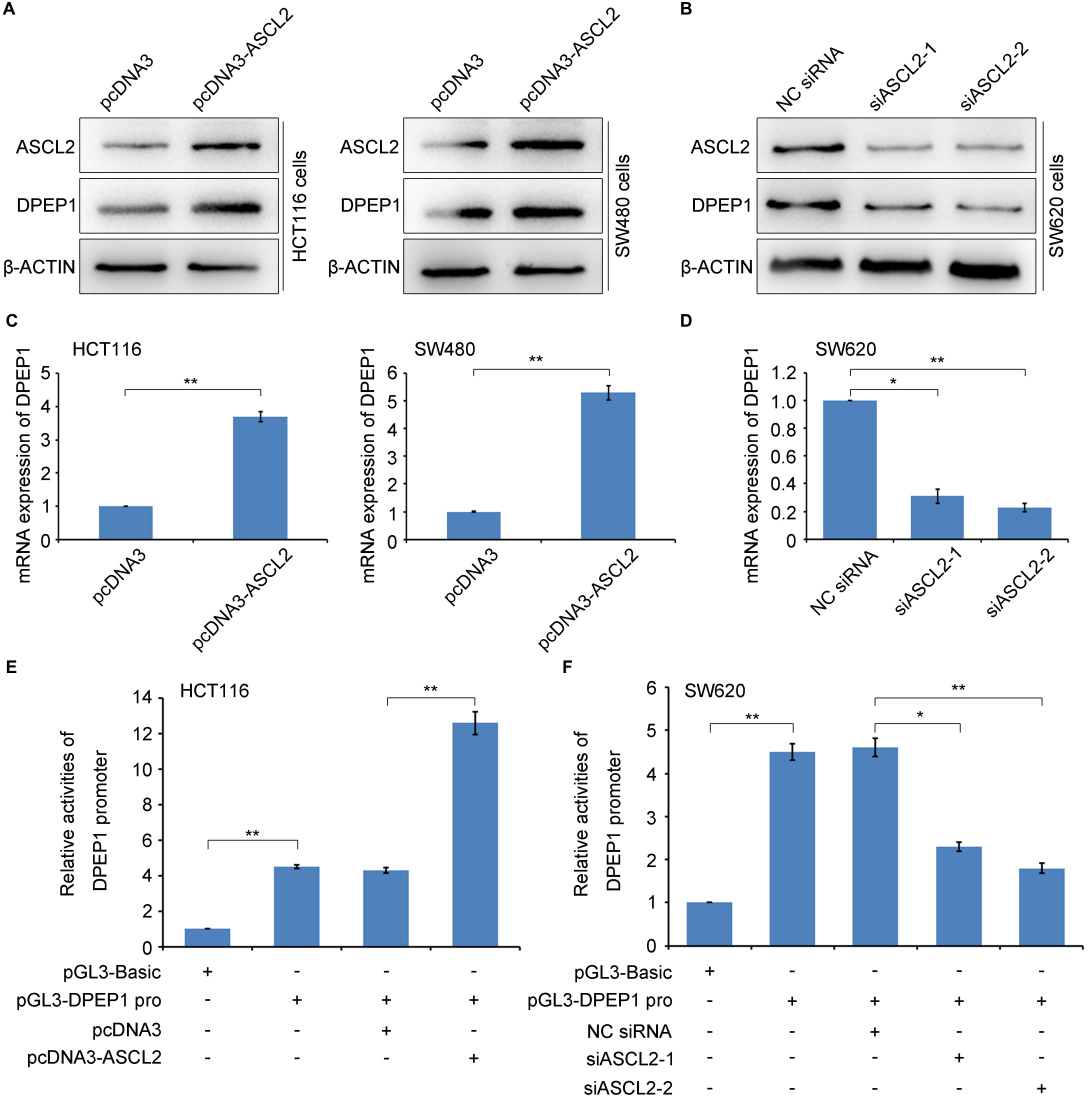
DPEP1 expression was upregulated by ASCL2 in colon cancer cells. (A) Western blotting was used to detect the protein levels of DPEP1 and ASCL2 in HCT116 and SW480 cells transiently transfected with pcDNA3 (0.8 μg) or pcDNA3‐ASCL2 (0.8 μg) plasmids, respectively. (B) Western blotting was used to detect the protein levels of DPEP1 and ASCL2 in SW620 cells that were transiently transfected with NC siRNA (100 nM), ASCL2 siRNA‐1 (100 nM), or ASCL2 siRNA‐2 (100 nM). (C) The mRNA expression level of DPEP1 was tested by qRT–PCR in HCT116 and SW480 cells that were transiently transfected with pcDNA3 (0.8 μg) or pcDNA3‐ASCL2 (0.8 μg) plasmids. (D) The mRNA expression level of DPEP1 was tested by qRT–PCR in SW620 cells transiently transfected with NC siRNA (100 nM), ASCL2 siRNA‐1 (100 nM), or ASCL2 siRNA‐2 (100 nM). (E) Relative activities of the DPEP1 promoter were detected by dual‐luciferase reporter experiments in HCT116 cells that were transiently transfected with pcDNA3 (0.8 μg) or pcDNA3‐ASCL2 (0.8 μg) plasmids. (F) Relative activities of the DPEP1 promoter were detected by dual‐luciferase reporter experiments in SW620 cells that were transiently transfected with NC siRNA (100 nM), ASCL2 siRNA‐1 (100 nM), or ASCL2 siRNA‐2 (100 nM). ASCL2, Achaete scute‐like 2; DPEP1, dipeptidase 1; NC, negative control. * represents *p* < 0.05; ** represents *p* < 0.01.

Given most reports indicated that ASCL2 was involved in the development of colon cancer as a transcription factor, we first explored whether ASCL2 regulated the expression of DPEP1. HCT116 and SW480 cells were transiently transfected with pcDNA3 or pcDNA3‐ASCL2 plasmids, and SW620 cells were transiently transfected with NC siRNA, siASCL2‐1, or siASCL2‐2. The results demonstrated that an increase in ASCL2 boosted the expression of DPEP1 (Figure 4A), and conversely, knockdown of ASCL2 notably decreased DPEP1 expression (Figure 4B). Similarly, ASCL2 heightened DPEP1 gene expression at the transcriptional level (Figure 4C,D). To further investigate whether ASCL2 regulates DPEP1 gene promoter activity, we constructed DPEP1 gene promoter into the pGL3‐basic plasmids. Dual‐luciferase reporter assays were performed to measure whether the transcription activity of the DPEP1 gene was regulated by ASCL2. The results displayed that the activities of the DPEP1 promoter were upregulated in the ASCL2‐dependent manner in HCT116 cells (Figure 4E), and its activity reduced when ASCL2 was knocked down in SW620 cells (Figure 4F). Generally, ASCL2 reinforces the expression of the DPEP1 gene by activating its promoter activity in the cells.

### 3.5 DPEP1 intensified drug resistance of colon cancer cells in an ASCL2‐dependent manner

**FIGURE 5:**
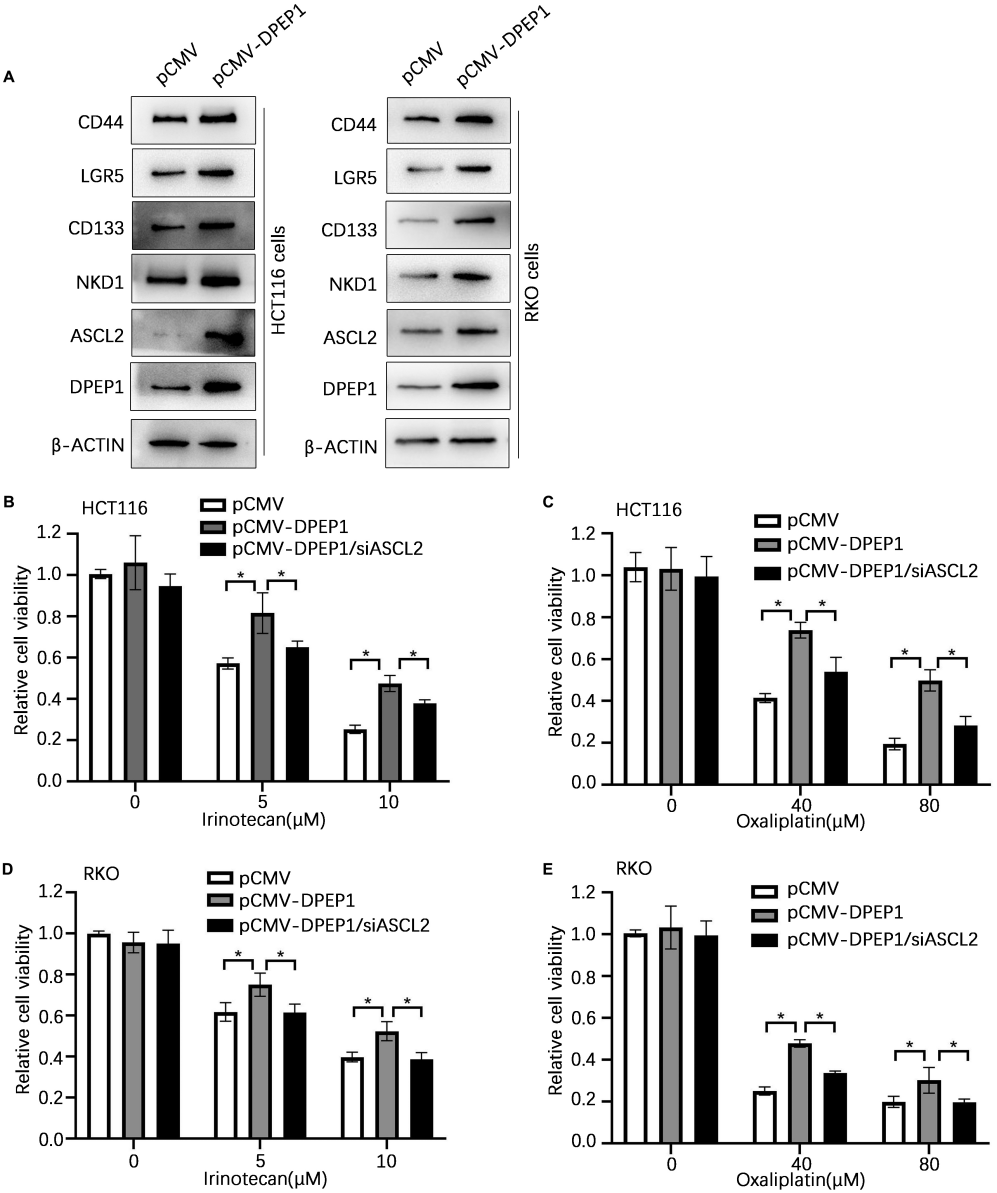
DPEP1 improves colon cancer cells chemoresistance in an ASCL2‐dependent manner. (A) Western blot analysis of DPEP1 and cell stem‐related proteins (ASCL2, CD44, CD133, and LGR5) in HCT116 and RKO cells. pCMV, pCMV‐DPEP1, and pCMV‐DPEP1 + siASCL2 were transiently transfected into HCT116 cells, and then each group of cells was treated with 0, 5, or 10 μM irinotecan (B) or 0, 40, or 80 μM oxaliplatin (C) for 48 h. pCMV, pCMV‐DPEP1, and pCMV‐DPEP1 + siASCL2 were transiently transfected into RKO cells, and then each group of cells was treated with 0, 5, or 10 μM irinotecan (D) or 0, 40, or 80 μM oxaliplatin (E) for 48 h. Cell viability was measured using MTT. ASCL2, Achaete scute‐like 2; DPEP1, dipeptidase 1. * represents *p* < 0.05.

Previous studies have shown that ASCL2 is not only a transcription factor, but also a marker of colon cancer stem cells.^23^, ^29^ We then wondered whether DPEP1 could regulate other colon cancer stem cell markers, such as ASCL2, CD133,^30^ CD44,^31^ and LGR5.^32^ We overexpressed DPEP1 in HCT116 and RKO cells, respectively, and then detected the expression of other colon cancer stem cell markers by western blotting, which demonstrated that the expression levels of ASCL2, CD133, CD44, LGR5, and NKD1 were markedly increased in the cells transfected with pCMV‐DPEP1 relative to the cells transfected with pCMV (Figure 5A), which suggested that DPEP1 could enhance the stemness of colon cancer cells. Given most reports indicated that drug resistance is an essential feature of cancer stem cells,^33^ and cell stemness is often closely related to drug resistance.^34^, ^35^ We then wondered whether DPEP1 could intensify the drug resistance of colon cancer cells. HCT116 and RKO cells transiently transfected respectively with pCMV, pCMV‐DPEP1, or pCMV‐DPEP1 + siASCL2 were measured by western blotting, which showed that DPEP1 overexpression inhibited the death of HCT116 and RKO cells that were incubated with irinotecan or oxaliplatin, whereas knockdown of ASCL2 significantly reversed this phenomenon (Figure 5B–E). Overall, these results reveal that DPEP1 intensifies the drug resistance of tumor cells in an ASCL2‐dependent manner by enhancing the stemness of colon cancer cells.

## 4 DISCUSSION

Colon cancer is the third death‐related disease in the world. Chemotherapy is the main way to treat cancer at present, and tumor drug resistance is the main obstacle to tumor treatment. However, the genes currently used for clinical diagnosis of colon tumor resistance are not only a few in number, but also low in specificity. In this study, we screened the massive gene data in the two databases through bioinformatics methods and finally obtained the DPEP1 gene. Increasing evidence has suggested that DPEP1 is a DEG in various tumors, and the biological function of DPEP1 in multiple tumors is still controversial. For example, DPEP1 is reduced in pancreatic ductal adenocarcinoma, inhibits the invasiveness of tumor cells, enhances chemosensitivity, and predicts a better clinical outcome.^36^ Moreover, DPEP1 acts as a tumor suppressor gene in breast cancer.^37^ In contrast, DPEP1 is highly expressed in leukemia cells, hepatoblastoma cells, and colon cancer cells and promotes tumor progression,^5^, ^6^, ^9^, ^10^, ^11^ suggesting that DPEP1 might act as an oncogene in these cancers. This study showed that DPEP1 was highly expressed in colon cancer tissues, which was consistent with previous studies.^7^, ^10^, ^11^ Bioinformatics analysis found that DPEP1 expression correlated negatively with DSS in colon cancer patients but not with OS. However, PA Eisenach et al. found that the DPEP1 high‐expression group had worse OS.^7^ We speculated that this difference might be due to the sample size and statistical methods.

In this study, we screened out four possible genes (ASCL2, RNF43, LY6G6D, and AXIN2), whose expressions were all positively correlated with DPEP1. We chose to study the first‐ranked ASCL2 gene to detect its relationship with DPEP1, but this did not mean that the other three genes were meaningless, and these genes still need to be further studied for their correlation with DPEP1. Because the expression of DPEP1 and ASCL2 in colon cancer tissues showed a markedly positive correlation, we speculated that there was a mutual regulation between DPEP1 and ASCL2, such regulation including direct or indirect mutual regulation, and more and more studies have demonstrated that this positive feedback loop regulation is common in tumor cells.^38^, ^39^ This study will provide a potentially important colon tumor drug resistance marker for clinical practice, and this positive feedback loop regulation will also provide a new perspective for tumor research.

In conclusion, ASCL2 upregulated DPEP1 expression levels in colon cancer cells. In turn, DPEP1 expression restrained ASCL2 protein degradation by the ubiquitin‐proteasome pathway. Therefore, DPEP1/ASCL2 could form a positive feedback loop regulation to maintain high expression levels of DPEP1 and ASCL2 in colon cancer cells, which may explain why DPEP1 induced an increase in drug resistance in colon cancer cells in an ASCL2‐dependent manner.

## AUTHOR CONTRIBUTIONS

All authors participated in the conception and design of the study; protocol/project development—Jianhua Jin and Qian Liu; data collection or management—Cheng Zeng, Guoping Qi, Ying Shen, Wenjing Li, and Qi Zhu; data analysis—Cheng Zeng, Chunxia Yang, Jianzhong Deng, and Wenbin Lu; manuscript writing/editing—Cheng Zeng. All authors read and approved the paper. Cheng Zeng and Guoping Qi contributed equally to this work.

## CONFLICT OF INTEREST

The authors have no conflict of interest.

## ETHICS APPROVAL AND CONSENT TO PARTICIPATE

This study was approved by the Ethics Committee of Wujin Hospital affiliated with Jiangsu University. Informed consent was obtained from patients before the study.

## CONSENT FOR PUBLICATION

Not applicable.

## Supporting information


Figure S1

Figure S2

Figure S3

Table S1

Table S2

Table S3

Table S4

Table S5

Table S6

## Data Availability

DPEP1 expression profiles in TCGA and Genotype‐Tissue Expression (GTEx) clinical pan‐cancer data from the University of California, Santa Cruz (UCSC) Xena database (https://xenabrowser.net/datapages/), and The Gene Expression Omnibus (GEO) dataset (https://www.ncbi.nlm.nih.gov/geo/) are available by contacting the author.
