# Supplementary figures and images for: DPEP1 promotes drug resistance in colon cancer cells by forming a positive feedback loop with ASCL2
Source: Cancer Med. 2022 Jun 6;12(1):412–24. doi: 10.1002/cam4.4926 (PMC9844606; doi:10.1002/cam4.4926)

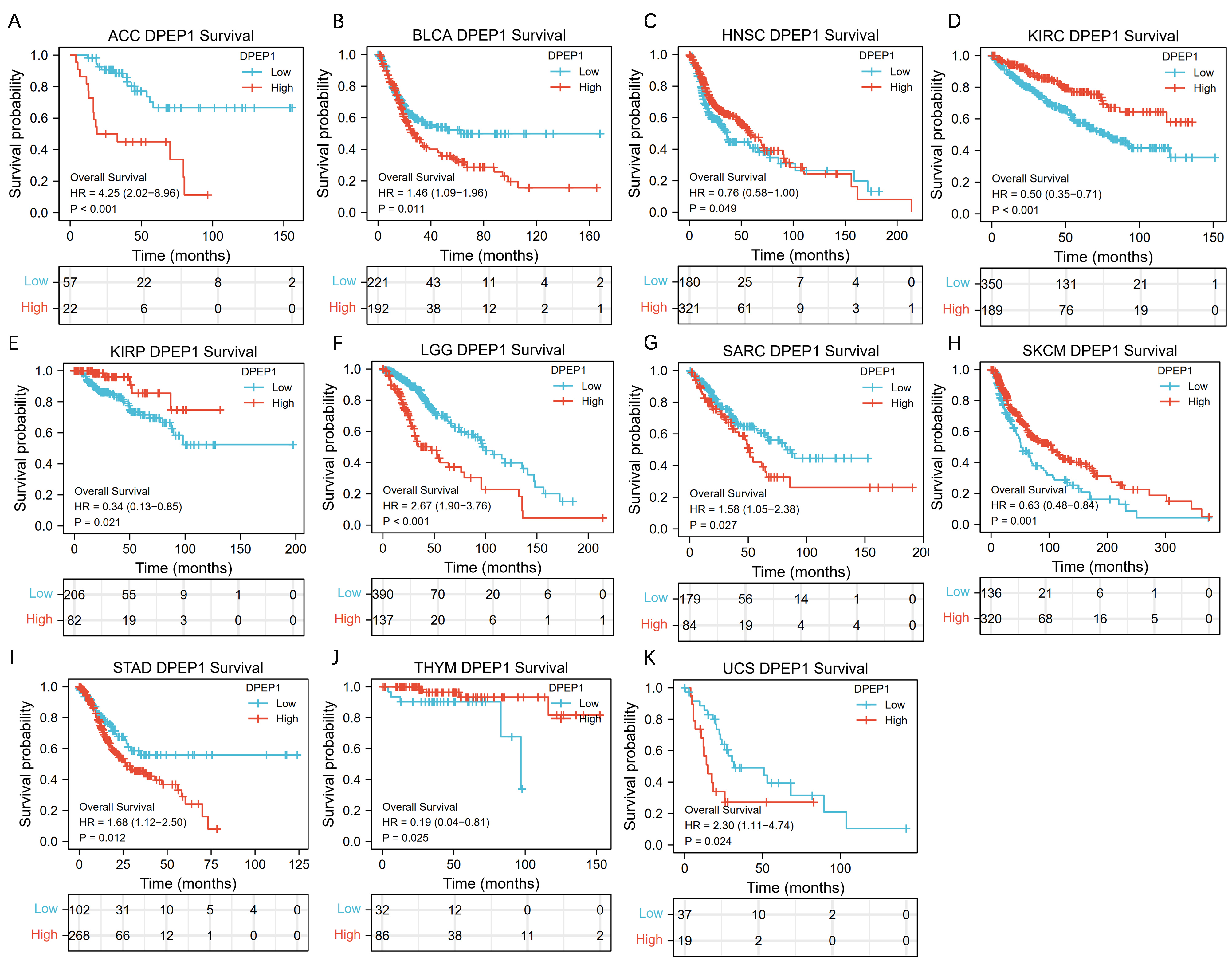

Supplement: Supplementary file 1 — Figure S1 [file CAM4-12-412-s004.jpg]

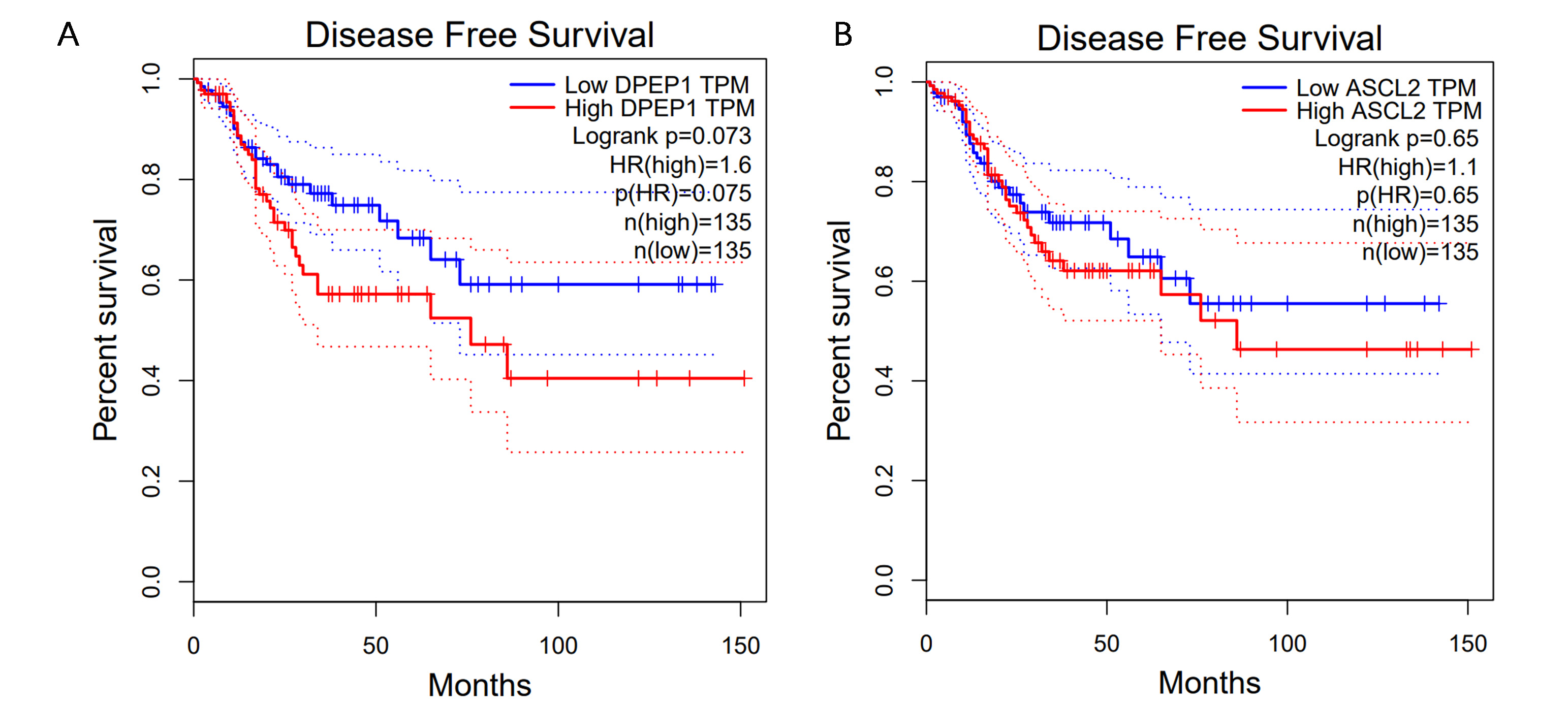

Supplement: Supplementary file 2 — Figure S2 [file CAM4-12-412-s007.jpg]

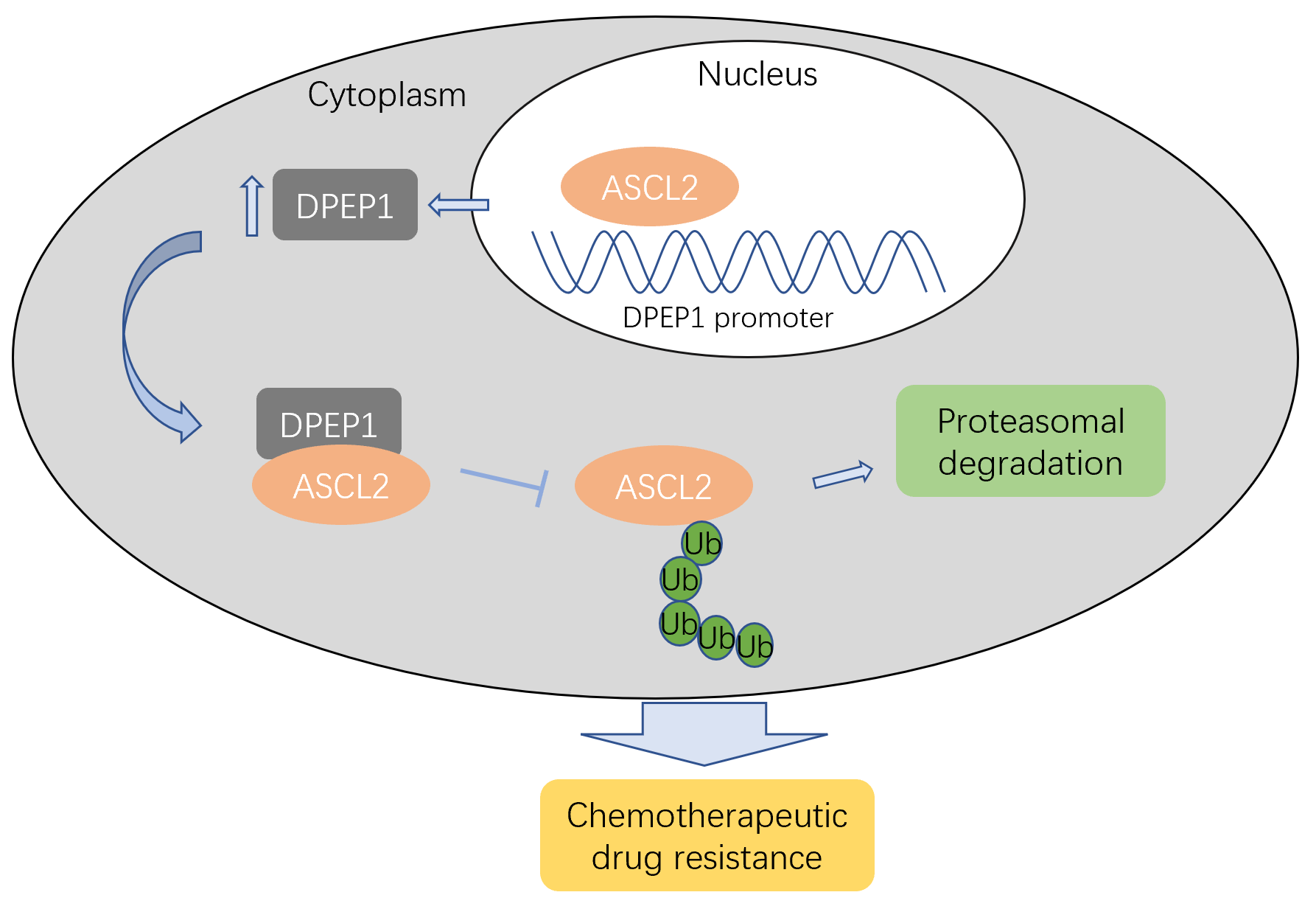

Supplement: Supplementary file 3 — Figure S3 [file CAM4-12-412-s001.png]
